# *N*′-(3,5-Dichloro-2-hy­droxy­benzyl­idene)-4-nitro­benzohydrazide methanol solvate

**DOI:** 10.1107/S1600536810036184

**Published:** 2010-09-15

**Authors:** Hai-Yun Zhu

**Affiliations:** a Department of Chemistry and Chemical Engineering, Baoji University of Arts and Sciences, Baoji 721013, People’s Republic of China

## Abstract

In the title compound, C_14_H_9_Cl_2_N_3_O_4_·CH_4_O, the dihedral angle between the two benzene rings in the hydrazone mol­ecule is 6.3 (3)°. An intra­molecular N—H⋯O hydrogen bond stabilizes the mol­ecular conformation. In the crystal, centrosymmetrically related mol­ecules are linked through inter­molecular O—H⋯O and N—H⋯O hydrogen bonds.

## Related literature

For general background to hydrazone compounds, see: Rasras *et al.* (2010 ▶); Fan *et al.* (2010 ▶); Ajani *et al.* (2010 ▶); Avaji *et al.* (2009 ▶). For the crystal structures of related hydrazone compounds, see: Khaledi *et al.* (2010 ▶); Han *et al.* (2010 ▶); Hussain *et al.* (2010 ▶); Ji & Lu (2010 ▶). For reference bond-length data, see: Allen *et al.* (1987 ▶).
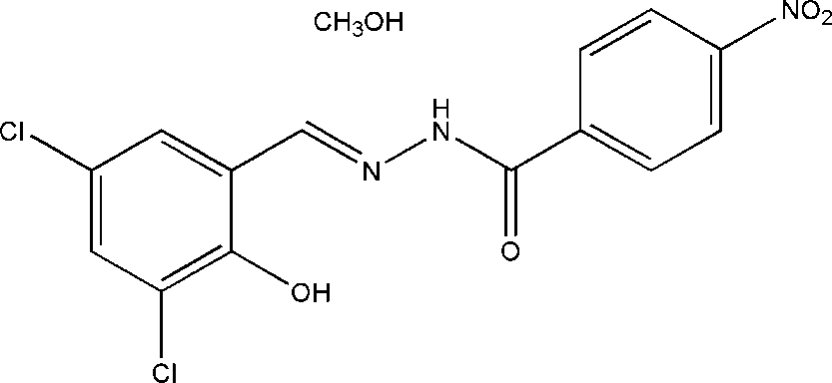


## Experimental

### 

#### Crystal data

C_14_H_9_Cl_2_N_3_O_4_·CH_4_O
*M* *_r_* = 386.18
Monoclinic, 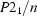

*a* = 7.415 (3) Å
*b* = 13.408 (3) Å
*c* = 16.674 (2) Å
β = 99.716 (3)°
*V* = 1634.0 (8) Å^3^
*Z* = 4
Mo *K*α radiation
μ = 0.43 mm^−1^
*T* = 298 K
0.15 × 0.13 × 0.10 mm

#### Data collection

Bruker SMART CCD area-detector diffractometer
Absorption correction: multi-scan (*SADABS*; Bruker, 2001 ▶) *T*_min_ = 0.938, *T*_max_ = 0.958
8410 measured reflections
3467 independent reflections
2099 reflections with *I* > 2σ(*I*)
*R*_int_ = 0.046

#### Refinement

*R*[*F* ^2^ > 2σ(*F* ^2^)] = 0.054
*wR*(*F* ^2^) = 0.124
*S* = 1.02
3467 reflections
232 parameters
1 restraint
H atoms treated by a mixture of independent and constrained refinement
Δρ_max_ = 0.21 e Å^−3^
Δρ_min_ = −0.31 e Å^−3^

### 

Data collection: *SMART* (Bruker, 2007 ▶); cell refinement: *SAINT* (Bruker, 2007 ▶); data reduction: *SAINT*; program(s) used to solve structure: *SHELXTL* (Sheldrick, 2008 ▶); program(s) used to refine structure: *SHELXTL*; molecular graphics: *SHELXTL*; software used to prepare material for publication: *SHELXTL*.

## Supplementary Material

Crystal structure: contains datablocks global, I. DOI: 10.1107/S1600536810036184/rz2485sup1.cif

Structure factors: contains datablocks I. DOI: 10.1107/S1600536810036184/rz2485Isup2.hkl

Additional supplementary materials:  crystallographic information; 3D view; checkCIF report

## Figures and Tables

**Table 1:** Hydrogen-bond geometry (Å, °)

*D*—H⋯*A*	*D*—H	H⋯*A*	*D*⋯*A*	*D*—H⋯*A*
O1—H1⋯N1	0.82	1.92	2.633 (3)	145
N2—H2⋯O5	0.90 (1)	1.91 (1)	2.793 (3)	167 (3)
O5—H5⋯O2^i^	0.82	2.15	2.903 (3)	153

Symmetry code: (i) 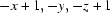.

**Fig. 1.**
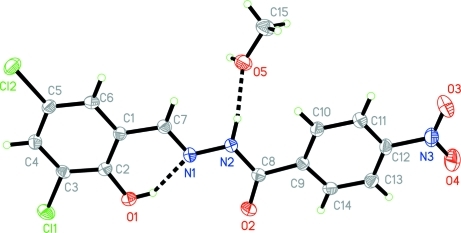
The molecular structure of the title compound with 30% probability displacement ellipsoids for non-hydrogen atoms. Hydrogen bonds are drawn as dashed lines.

**Fig. 2.**
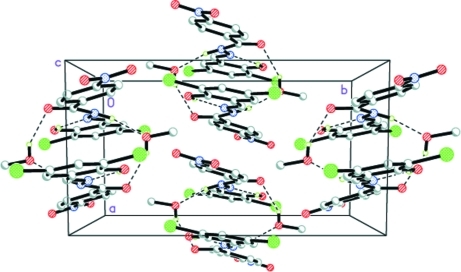
The molecular packing of the title compound viewed along the c axis. Hydrogen atoms not involved in hydrogen bonds (dashed lines) are omitted for clarity.

## supplementary crystallographic information

### Comment

In recent years, considerable interest has been focused on the preparation and biological properties of hydrazone compounds (Rasras *et al.*, 2010; Fan *et al.*, 2010; Ajani *et al.*, 2010; Avaji *et al.*, 2009). The crystal structures of a number of hydrazone compounds have been reported (Khaledi *et al.*, 2010; Han *et al.*, 2010; Hussain *et al.*, 2010; Ji & Lu, 2010). The author reports in this paper the title new hydrazone compound.

The asymmetric unit of the title compound (Fig. 1) consists of a hydrazone molecule and a methanol molecule. The dihedral angle between the C1—C6 and C9—C14 benzene rings is 6.3 (3)°. There is an intramolecular O—H···N hydrogen bond (Table 1) stabilizing the conformation of the hydrazone molecule. All bond lengths are within normal values (Allen *et al.*, 1987), and are comparable with those in the similar hydrazone compounds as cited above. In the crystal structure (Fig. 2), centrosymmetrically related molecules are linked through intermolecular O—H···O and N—H···O hydrogen bonds (Table 1).

### Experimental

3,5-Dichlorosalicylaldehyde (0.191 g, 1 mmol) and 4-nitrobenzohydrazide (0.181 g, 1 mmol) were dissolved in 30 ml absolute methanol. The mixture was stirred at reflux for 10 min, and cooled to room temperature. The clear yellow solution was left to slowly evaporate in air for a week, yielding yellow needle crystals of the title compound suitable for X-ray analysis.

### Refinement

The H2 atom attached to N2 was located in a difference Fourier map and refined isotropically, with the N—H distance restrained to 0.90 (1) Å, and with *U*_iso_ fixed at 0.08 Å^2^. The remaining H atoms were positioned geometrically and refined using the riding-model approximation, with C—H = 0.93–0.96 Å, O—H = 0.82 Å, and with *U*_iso_(H) = 1.2*U*_eq_(C) or 1.5*U*_eq_(C, O) for methyl and hydroxy H atoms.

### Crystal data

**Table:**

C_14_H_9_Cl_2_N_3_O_4_·CH_4_O	*F*(000) = 792
*M**_r_* = 386.18	*D*_x_ = 1.570 Mg m^−^^3^
Monoclinic, *P*2_1_/*n*	Mo *K*α radiation, λ = 0.71073 Å
Hall symbol: -P 2yn	Cell parameters from 1245 reflections
*a* = 7.415 (3) Å	θ = 2.4–24.5°
*b* = 13.408 (3) Å	µ = 0.43 mm^−^^1^
*c* = 16.674 (2) Å	*T* = 298 K
β = 99.716 (3)°	Cut from needle, yellow
*V* = 1634.0 (8) Å^3^	0.15 × 0.13 × 0.10 mm
*Z* = 4	

### Data collection

**Table:**

Bruker SMART CCD area-detector diffractometer	3467 independent reflections
Radiation source: fine-focus sealed tube	2099 reflections with *I* > 2σ(*I*)
graphite	*R*_int_ = 0.046
ω scans	θ_max_ = 27.0°, θ_min_ = 2.5°
Absorption correction: multi-scan (*SADABS*; Bruker, 2001)	*h* = −9→9
*T*_min_ = 0.938, *T*_max_ = 0.958	*k* = −14→17
8410 measured reflections	*l* = −21→17

### Refinement

**Table:**

Refinement on *F*^2^	Primary atom site location: structure-invariant direct methods
Least-squares matrix: full	Secondary atom site location: difference Fourier map
*R*[*F*^2^ > 2σ(*F*^2^)] = 0.054	Hydrogen site location: inferred from neighbouring sites
*wR*(*F*^2^) = 0.124	H atoms treated by a mixture of independent and constrained refinement
*S* = 1.02	*w* = 1/[σ^2^(*F*_o_^2^) + (0.0478*P*)^2^ + 0.0676*P*] where *P* = (*F*_o_^2^ + 2*F*_c_^2^)/3
3467 reflections	(Δ/σ)_max_ < 0.001
232 parameters	Δρ_max_ = 0.21 e Å^−^^3^
1 restraint	Δρ_min_ = −0.31 e Å^−^^3^

### Special details

**Table:**

Geometry. All e.s.d.'s (except the e.s.d. in the dihedral angle between two l.s. planes) are estimated using the full covariance matrix. The cell e.s.d.'s are taken into account individually in the estimation of e.s.d.'s in distances, angles and torsion angles; correlations between e.s.d.'s in cell parameters are only used when they are defined by crystal symmetry. An approximate (isotropic) treatment of cell e.s.d.'s is used for estimating e.s.d.'s involving l.s. planes.
Refinement. Refinement of *F*^2^ against ALL reflections. The weighted *R*-factor *wR* and goodness of fit *S* are based on *F*^2^, conventional *R*-factors *R* are based on *F*, with *F* set to zero for negative *F*^2^. The threshold expression of *F*^2^ > σ(*F*^2^) is used only for calculating *R*-factors(gt) *etc*. and is not relevant to the choice of reflections for refinement. *R*-factors based on *F*^2^ are statistically about twice as large as those based on *F*, and *R*- factors based on ALL data will be even larger.

### Fractional atomic coordinates and isotropic or equivalent isotropic displacement parameters (Å^2^)

**Table:**

	*x*	*y*	*z*	*U*_iso_*/*U*_eq_	
Cl1	0.46614 (12)	−0.21402 (6)	0.15195 (5)	0.0565 (3)	
Cl2	0.37115 (12)	0.17025 (6)	0.06479 (5)	0.0538 (3)	
N1	0.2955 (3)	−0.01141 (17)	0.40697 (13)	0.0378 (6)	
N2	0.2663 (3)	0.02375 (17)	0.48171 (14)	0.0388 (6)	
N3	0.0804 (4)	0.1043 (3)	0.84113 (17)	0.0567 (8)	
O1	0.3717 (3)	−0.14918 (14)	0.30523 (12)	0.0481 (6)	
H1	0.3572	−0.1275	0.3497	0.072*	
O2	0.2382 (3)	−0.13351 (15)	0.52776 (12)	0.0552 (6)	
O3	0.0317 (4)	0.1905 (2)	0.84516 (15)	0.0786 (8)	
O4	0.1004 (4)	0.0463 (2)	0.89825 (15)	0.0911 (9)	
O5	0.4237 (3)	0.21205 (16)	0.51288 (15)	0.0619 (7)	
H5	0.5275	0.2100	0.5021	0.093*	
C1	0.3366 (4)	0.0260 (2)	0.27209 (16)	0.0329 (6)	
C2	0.3712 (4)	−0.0733 (2)	0.25280 (16)	0.0356 (7)	
C3	0.4095 (4)	−0.0933 (2)	0.17535 (16)	0.0349 (7)	
C4	0.4077 (4)	−0.0197 (2)	0.11735 (17)	0.0410 (7)	
H4	0.4311	−0.0353	0.0657	0.049*	
C5	0.3705 (4)	0.0773 (2)	0.13695 (16)	0.0370 (7)	
C6	0.3342 (4)	0.1002 (2)	0.21322 (16)	0.0363 (7)	
H6	0.3079	0.1656	0.2256	0.044*	
C7	0.3046 (4)	0.0552 (2)	0.35267 (16)	0.0373 (7)	
H7	0.2908	0.1223	0.3647	0.045*	
C8	0.2371 (4)	−0.0435 (2)	0.53888 (17)	0.0369 (7)	
C9	0.1972 (4)	−0.0003 (2)	0.61643 (16)	0.0332 (7)	
C10	0.1438 (4)	0.0976 (2)	0.62461 (17)	0.0395 (7)	
H10	0.1332	0.1406	0.5803	0.047*	
C11	0.1061 (4)	0.1320 (2)	0.69819 (18)	0.0440 (8)	
H11	0.0710	0.1978	0.7040	0.053*	
C12	0.1216 (4)	0.0669 (2)	0.76247 (17)	0.0405 (7)	
C13	0.1716 (4)	−0.0309 (2)	0.75640 (17)	0.0439 (8)	
H13	0.1796	−0.0737	0.8008	0.053*	
C14	0.2096 (4)	−0.0643 (2)	0.68295 (17)	0.0400 (7)	
H14	0.2439	−0.1304	0.6777	0.048*	
C15	0.3910 (5)	0.3073 (2)	0.5430 (2)	0.0592 (9)	
H15A	0.4591	0.3147	0.5970	0.089*	
H15B	0.2628	0.3147	0.5444	0.089*	
H15C	0.4288	0.3574	0.5082	0.089*	
H2	0.300 (5)	0.0871 (11)	0.494 (2)	0.080*	

### Atomic displacement parameters (Å^2^)

**Table:**

	*U*^11^	*U*^22^	*U*^33^	*U*^12^	*U*^13^	*U*^23^
Cl1	0.0704 (6)	0.0374 (5)	0.0641 (6)	0.0041 (4)	0.0181 (4)	−0.0126 (4)
Cl2	0.0633 (6)	0.0514 (5)	0.0479 (5)	−0.0048 (4)	0.0127 (4)	0.0148 (4)
N1	0.0435 (15)	0.0393 (14)	0.0321 (13)	−0.0018 (12)	0.0109 (11)	−0.0040 (11)
N2	0.0501 (16)	0.0345 (14)	0.0334 (13)	−0.0035 (13)	0.0120 (11)	−0.0031 (11)
N3	0.0562 (19)	0.072 (2)	0.0457 (18)	−0.0123 (17)	0.0203 (14)	−0.0134 (16)
O1	0.0704 (16)	0.0325 (12)	0.0429 (12)	−0.0015 (11)	0.0138 (12)	0.0040 (9)
O2	0.0864 (18)	0.0301 (12)	0.0527 (14)	−0.0008 (12)	0.0223 (12)	−0.0067 (10)
O3	0.098 (2)	0.076 (2)	0.0684 (18)	0.0053 (17)	0.0328 (15)	−0.0264 (15)
O4	0.143 (3)	0.093 (2)	0.0445 (15)	−0.0060 (19)	0.0359 (16)	−0.0005 (15)
O5	0.0673 (17)	0.0488 (14)	0.0767 (17)	−0.0132 (12)	0.0326 (14)	−0.0180 (12)
C1	0.0328 (16)	0.0336 (16)	0.0335 (15)	−0.0002 (13)	0.0089 (12)	−0.0015 (12)
C2	0.0350 (17)	0.0346 (17)	0.0375 (16)	−0.0021 (13)	0.0065 (13)	0.0027 (13)
C3	0.0342 (17)	0.0311 (16)	0.0406 (16)	0.0007 (13)	0.0094 (13)	−0.0066 (13)
C4	0.0444 (19)	0.0441 (18)	0.0354 (16)	−0.0056 (15)	0.0095 (13)	−0.0027 (14)
C5	0.0375 (17)	0.0383 (17)	0.0357 (16)	−0.0036 (14)	0.0081 (13)	0.0039 (13)
C6	0.0371 (17)	0.0290 (15)	0.0423 (17)	0.0011 (13)	0.0056 (13)	0.0008 (13)
C7	0.0401 (18)	0.0323 (16)	0.0406 (16)	0.0026 (14)	0.0099 (13)	−0.0024 (13)
C8	0.0384 (18)	0.0350 (17)	0.0378 (16)	−0.0028 (14)	0.0078 (13)	−0.0025 (14)
C9	0.0307 (16)	0.0351 (16)	0.0344 (15)	−0.0016 (13)	0.0075 (12)	−0.0028 (13)
C10	0.0459 (19)	0.0368 (17)	0.0387 (17)	0.0034 (14)	0.0157 (14)	0.0019 (13)
C11	0.048 (2)	0.0404 (18)	0.0461 (18)	0.0010 (15)	0.0150 (15)	−0.0044 (15)
C12	0.0340 (17)	0.054 (2)	0.0356 (16)	−0.0081 (15)	0.0132 (13)	−0.0094 (15)
C13	0.048 (2)	0.047 (2)	0.0368 (17)	−0.0054 (16)	0.0068 (14)	0.0039 (14)
C14	0.0417 (18)	0.0332 (17)	0.0458 (18)	−0.0059 (14)	0.0094 (14)	−0.0009 (14)
C15	0.058 (2)	0.047 (2)	0.074 (2)	0.0044 (17)	0.0156 (18)	−0.0058 (18)

### Geometric parameters (Å, °)

**Table:**

Cl1—C3	1.733 (3)	C4—C5	1.380 (4)
Cl2—C5	1.733 (3)	C4—H4	0.9300
N1—C7	1.282 (3)	C5—C6	1.378 (4)
N1—N2	1.383 (3)	C6—H6	0.9300
N2—C8	1.356 (4)	C7—H7	0.9300
N2—H2	0.898 (10)	C8—C9	1.492 (4)
N3—O3	1.217 (4)	C9—C10	1.385 (4)
N3—O4	1.219 (3)	C9—C14	1.393 (4)
N3—C12	1.483 (4)	C10—C11	1.383 (4)
O1—C2	1.341 (3)	C10—H10	0.9300
O1—H1	0.8200	C11—C12	1.372 (4)
O2—C8	1.221 (3)	C11—H11	0.9300
O5—C15	1.409 (3)	C12—C13	1.371 (4)
O5—H5	0.8200	C13—C14	1.377 (4)
C1—C6	1.396 (4)	C13—H13	0.9300
C1—C2	1.403 (4)	C14—H14	0.9300
C1—C7	1.457 (4)	C15—H15A	0.9600
C2—C3	1.394 (4)	C15—H15B	0.9600
C3—C4	1.380 (4)	C15—H15C	0.9600
			
C7—N1—N2	115.7 (2)	C1—C7—H7	120.0
C8—N2—N1	118.3 (2)	O2—C8—N2	123.0 (3)
C8—N2—H2	123 (2)	O2—C8—C9	121.5 (3)
N1—N2—H2	116 (2)	N2—C8—C9	115.4 (2)
O3—N3—O4	124.2 (3)	C10—C9—C14	119.1 (3)
O3—N3—C12	118.5 (3)	C10—C9—C8	123.7 (3)
O4—N3—C12	117.3 (3)	C14—C9—C8	117.1 (3)
C2—O1—H1	109.5	C11—C10—C9	120.5 (3)
C15—O5—H5	109.5	C11—C10—H10	119.8
C6—C1—C2	119.7 (2)	C9—C10—H10	119.8
C6—C1—C7	118.2 (3)	C12—C11—C10	118.6 (3)
C2—C1—C7	122.1 (3)	C12—C11—H11	120.7
O1—C2—C3	118.6 (3)	C10—C11—H11	120.7
O1—C2—C1	123.4 (3)	C13—C12—C11	122.6 (3)
C3—C2—C1	118.0 (2)	C13—C12—N3	119.2 (3)
C4—C3—C2	122.1 (3)	C11—C12—N3	118.2 (3)
C4—C3—Cl1	119.0 (2)	C12—C13—C14	118.4 (3)
C2—C3—Cl1	118.9 (2)	C12—C13—H13	120.8
C5—C4—C3	119.1 (3)	C14—C13—H13	120.8
C5—C4—H4	120.4	C13—C14—C9	120.8 (3)
C3—C4—H4	120.4	C13—C14—H14	119.6
C6—C5—C4	120.4 (3)	C9—C14—H14	119.6
C6—C5—Cl2	120.3 (2)	O5—C15—H15A	109.5
C4—C5—Cl2	119.3 (2)	O5—C15—H15B	109.5
C5—C6—C1	120.6 (3)	H15A—C15—H15B	109.5
C5—C6—H6	119.7	O5—C15—H15C	109.5
C1—C6—H6	119.7	H15A—C15—H15C	109.5
N1—C7—C1	120.0 (3)	H15B—C15—H15C	109.5
N1—C7—H7	120.0		

### Hydrogen-bond geometry (Å, °)

**Table:**

*D*—H···*A*	*D*—H	H···*A*	*D*···*A*	*D*—H···*A*
O1—H1···N1	0.82	1.92	2.633 (3)	145
N2—H2···O5	0.90 (1)	1.91 (1)	2.793 (3)	167 (3)
O5—H5···O2^i^	0.82	2.15	2.903 (3)	153

Symmetry codes: (i) −*x*+1, −*y*, −*z*+1.
